# Assessment of the accuracy of biparametric MRI/TRUS fusion-guided biopsy for index tumor evaluation using postoperative pathology specimens

**DOI:** 10.1186/s12894-024-01473-0

**Published:** 2024-04-04

**Authors:** Ryutaro Shimizu, Shuichi Morizane, Atsushi Yamamoto, Hiroshi Yamane, Ryoma Nishikawa, Yusuke Kimura, Noriya Yamaguchi, Katsuya Hikita, Masashi Honda, Atsushi Takenaka

**Affiliations:** Division of Urology, Department of Surgery, Faculty of Medicine, Tottori University, 36-1, Nishi-cho, Yonago, 683-8504 Japan

**Keywords:** Prostate cancer, Biparametric MRI, MRI/transrectal ultrasound fusion prostate biopsy

## Abstract

**Background:**

Multiparametric MRI (mpMRI) is widely used for the diagnosis, surveillance, and staging of prostate cancer. However, it has several limitations, including higher costs, longer examination times, and the use of gadolinium-based contrast agents. This study aimed to investigate the accuracy of preoperatively assessed index tumors (ITs) using biparametric MRI (bpMRI)/transrectal ultrasound (TRUS) fusion biopsy compared with radical prostatectomy (RP) specimens.

**Methods:**

We included 113 patients diagnosed with prostate cancer through bpMRI/TRUS fusion-guided biopsies of lesions with a Prostate Imaging Reporting and Data System (PI-RADS) category ≥ 3. These patients underwent robot-assisted laparoscopic radical prostatectomy (RARP) at our institution between July 2017 and March 2023. We examined the localization of preoperative and postoperative ITs, the highest Gleason score (GS), and tumor diameter in these patients.

**Results:**

The preoperative cT stage matched the postoperative pT stage in 53 cases (47%), while 31 cases (27%) were upstaged, and 29 cases (26%) were downstaged (Weighted Kappa = 0.21). The preoperative and postoperative IT localizations were consistent in 97 cases (86%). The concordance rate between Gleason groups in targeted biopsies and RP specimens was 51%, with an upgrade in 25 cases (23%) and a downgrade in 27 cases (25%) (Weighted Kappa = 0.42). The maximum diameter of the IT and the maximum cancer core length on biopsy were correlated with the RP tumor's maximum diameter (*p* < 0.001 for both).

**Conclusion:**

The diagnostic accuracy of bpMRI/TRUS fusion biopsy is comparable to mpMRI, suggesting that it can be a cost-effective and time-saving alternative.

## Introduction

Prostate cancer (PCa) diagnosis is primarily based on prostate-specific antigen (PSA), imaging, and histology results. Based on this diagnostic information, a risk classification is established, and treatment is determined. However, issues such as image quality and biopsy sampling errors can lead to diagnostic inaccuracies. Especially in prostate cancer surgery, we often experience discrepancies between the preoperative evaluation and the radical prostatectomy specimen. Prostate cancer surgery, including nerve sparing and lymph node dissection, is planned based on preoperative assessments of lesion localization and grade using MRI images and biopsy results. However, there are cases in which preoperative assessments underestimate the extent of cancer, leading to positive margins in postoperative pathology, or overestimate it, resulting in missed opportunities for nerve-sparing [1, 2].

Multiparametric MRI (mpMRI) is a widely used technique for prostate cancer diagnosis, surveillance, and staging [3]. Accurate tumor localization with mpMRI and fusion of MR and transrectal ultrasound (TRUS) images for biopsy may provide a more accurate preoperative evaluation [4]. This technique, known as mpMRI/TRUS fusion-guided biopsy, has become increasingly common, and its accuracy has been validated in several studies using postoperative pathology specimens [5, 6]. However, mpMRI has limitations, including higher cost, longer examination time, and the use of gadolinium-based contrast agents. Our institution uses biparametric MRI (bpMRI), including the T2W and DW MRI series, for the diagnosis of prostate cancer. Several studies have shown that bpMRI provides similar results to mpMRI in detecting and localizing PCa [7, 8]. However, no studies have compared bpMRI with radical prostatectomy (RP) specimens.

In the present study, we examined the accuracy of index tumor (IT) assessed preoperatively using bpMRI-TRUS fusion prostate biopsy with RP specimens.

## Materials and methods

This study was approved by the Ethics Committee of Tottori University Faculty of Medicine, Yonago, Japan (approval number: 20A016), and was conducted in accordance with the ethical guidelines set by the government. As this study involved only medical data and did not involve direct patient contact, informed consent was waived by the ethics committee. The study details were disclosed on the website in advance to ensure transparency and adherence to ethical standards.

### Patients

Between July 2017 and March 2023, out of 404 patients who underwent robotic-assisted laparoscopic radical prostatectomy (RARP) at our institution, 113 were included in this study. These patients were diagnosed with prostate cancer through bpMRI/TRUS fusion-guided biopsy of lesions with a Prostate Imaging Reporting and Data System (PI-RADS) category ≥ 3. The remaining 291 patients were excluded because they were diagnosed without bpMRI/TRUS fusion-guided biopsy or were diagnosed at other hospitals.

### MRI and registration analysis

At our institution, mpMRI is generally not performed for the initial diagnosis of prostate cancer due to considerations of time and bpMRI is utilized. All male patients underwent a 1.5T or 3T bpMRI. PI-RADS guidelines state that both 1.5T and 3.0T can provide adequate and reliable diagnostic examinations [9]. MRI imaging conditions were in accordance with the PI-RADS [9]. Prior to biopsy, all suspicious lesions found on prostate MRI were scored by a single board-certified radiologist with expertise in prostate imaging. If MRI was initially conducted and read by a third-party radiologist, a second reading was performed at our institution, and scoring was based on the PI-RADS guideline recommendations. Additionally, during the imaging evaluation, the radiologist was informed about patients’ PSA levels, age, and other clinical information.

Preoperative IT localization, highest Gleason score (GS), and tumor diameter were examined in these patients. The tumor area was determined by the radiologist using a sector map adapted from the European Consensus Conference and ESUR Prostate MRI Guidelines 2012 to PI-RADS v2 [10]. For this study, preoperative IT was defined as a positive target biopsy with a PI-RADS category ≥ 3 and the largest lesion. Clinical T staging was based on lesions with PI-RADS ≥ 4, or PI-RADS 3 lesions identified as significant due to positive target biopsy findings.

### Prostate biopsy

The TRINITYTM system (Koelis, La Tronche, France) was utilized for all biopsy procedures, with the patient under spinal epidural anesthesia in the lithotripsy position. Initially, we visualized three-dimensional (3D) volume data obtained from MRI and real-time TRUS images. Elastic image fusion was conducted by semi-automatically contouring the MRI image of the entire prostate and suspected lesions on 3D TRUS images. The biopsy procedure involved a two-core biopsy targeted to each suspicious lesion identified on MRI, followed by a 10–14 core systematic biopsy (SB). If there were three or more MRI lesions, two MRI-targeted biopsies (TBs) were performed. In this case, one MRI-TB was conducted on the index lesion and another on the next suspected lesion. All biopsy cores were obtained by experienced urologists (T.S., S.R.).

### Surgery and pathology for registration analysis

Robotic RP was performed on all patients, and the surgical specimens were fixed in 10% neutral-buffered formalin, embedded in paraffin blocks, and stained with hematoxylin and eosin. The inferior-most 5–7-mm portion of the gland was defined as the apex of the prostate, while the superior-most 5–7-mm portion of the gland was defined as the base of the prostate; the remainder was defined as the mid-gland. The apex and base of the prostate were divided into sections ranging from 3–5 mm in sagittal planes, and the mid-gland was sectioned into 3–5 mm in horizontal planes. IT localization was derived from pathology reports prepared by pathologists. In RP specimens, an IT lesion was defined either as a lesion with extraprostatic extension or as the lesion with the largest volume. Regular meetings were held among urologists, radiologists, and pathologists to discuss and verify the consistency of the IT positioning. To assess concordance rates for pathology assessment, we utilized the revised prostate cancer grading system, Grade Group (GG), released by the International Society of Urologic Pathology (ISUP) in 2014 [11].

### Statistical analyses

A t-test was used to evaluate the difference between MR-estimated IT diameter and histological-IT diameter. To assess agreement between biopsies and RP specimens in GG, as well as between cTstage and pTstage, we used weighted Kappa statistics (k). For all tests, *P* values < 0.05, were considered statistically significant. Statistical analyses were conducted using SPSS Statistics software version 24.0 (SPSS Inc.,).

## Results

### Patients

Table 1 displays the patient characteristics before prostate biopsy. The mean age of the 113 patients with preoperative IT was 71 years (interquartile range: 66–74 years). The median PSA was 8.7 ng/ml (interquartile range: 5.7–12.1 ng/ml) and the median prostate volume was 28.9 ml (interquartile range: 22.5–37.1 ml). The median waiting time from biopsy to surgery was 122 days (interquartile range: 99.5–160.5). MRI was conducted at 3.0 T and 1.5 T in 96 and 17 patients, respectively. The PI-RADS rating of IT on pre-biopsy MRI evaluation was 3, 4, and 5 in 20 (18%), 58 (51%), and 35 patients (31%), respectively.

**Table 1:** Patient characteristics

Age, median (interquartile range)		71 (66–74)
PSA, ng/ml, median (interquartile range)		8.7 (5.7–12.1)
prostate volume, ml, median (interquartile range)		28.9 (22.5–37.1)
median waiting time, day, median (interquartile range)		122 (100–161)
Pre-operative IT	PI-RADS 3, n (%)	20 (18%)
	PI-RADS 4, n (%)	58 (51%)
	PI-RADS 5, n (%)	35 (31%)
cT stage	cT2a, n (%)	32 (28%)
	cT2b, n (%)	1 (0.9%)
	cT2c, n (%)	38 (34%)
	cT3a, n (%)	41 (36%)
	cT3b, n (%)	1 (1.4%)

*Abbreviations*: *PSA* prostate-specific antigen

### Accuracy of bpMRI Tstage diagnosis

The distribution of cTstage was as follows: T2a in 32 cases (28%), T2b in 1 case (0.9%), T2c in 38 cases (34%), T3a in 41 cases (36%), and T3b in 1 case (0.9%). The pTstage was T2a in 9 cases (8.0%), T2b in 2 cases (1.8%), T2c in 79 cases (70%), T3a in 21 cases (19%), and T3b in 2 cases (1.8%). Of the cTstage cases, 31 (27%) were underestimated, 29 (26%) were overestimated, and 53 (47%) were concordant, resulting in a weighted kappa coefficient of 0.22 (Table 2).

**Table 2:** Accuracy of MRI T-stage diagnosis

	pT2a	pT2b	pT2c	pT3a	pT3b	total
cT2a	7 (6.2%)	1 (0.9%)	19 (17%)	5 (4.4%)	0	32 (28%)
cT2b	0	0	1 (0.9%)	0	0	1 (0.9%)
cT2c	0	0	33 (29%)	3 (2.7%)	2 (1.8%)	38 (34%)
cT3a	1 (0.9%)	1 (0.9%)	26 (23%)	13 (12%)	0	41 (36%)
cT3b	1 (0.9%)	0	0	0	0	1 (0.9%)
Total	9 (8.0%)	2 (1.8%)	79 (67%)	21 (16%)	2 (1.8%)	113

### Accuracy of IT localization and size diagnosis

The agreement between the localization of IT assessed by bpMRI and biopsy with that in the RP specimen was 86% (Table 3). The diagnostic accuracy of IT localization was not affected by the magnet strengths of the MRI (1.5T or 3.0T) (*p* = 0.76). The mean diameter of IT assessed by bpMRI was 12.0 mm (interquartile range: 8.3–14.8 mm), while that in the RP specimen was 17.0 mm (interquartile range: 13.0 -22.8 mm).

**Table 3:** Accuracy of IT localization and radial margin (RM) positive rate

	Concordance	Discordance
Localization, n (%)	97 (86%)	16 (14%)
RM + , n/all (%)	21/97 (22%)	4/16 (25%)

*Abbreviation*: *RM* radial margin

### Grade Group (GG) concordance between biopsy and RP specimens

**Fig. 1:**
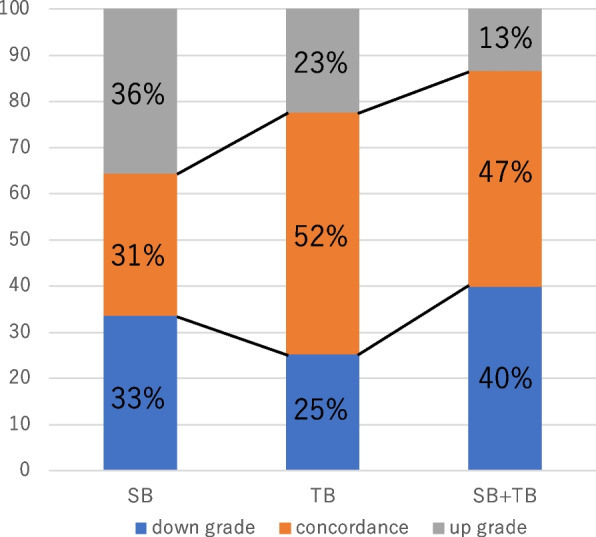
Relative rates of biopsy and radical prostatectomy specimen downgrading (blue bars), concordance (orange bars), and upgrading (gray bars) by systematic biopsy (SB), target biopsy (TB), and SB plus TB

Table 4 and Fig. 1 present the GG concordance between systematic biopsy (SB), targeted biopsy (TB), SB + TB, and RP specimens.

**Table 4:** Pathology concordance of biopsy schemes and radical prostatectomy specimen final pathology

		Radical prostatectomy specimens (GG)			
biopsy specimens	1	2	3	4	5
SB (*n* = 104)	0	33 (32%)	36 (35%)	13 (12%)	22 (21%)
GG1 (*n* = 14)	0	7 (6.7%)	7 (6.7%)	0	0
GG2 (*n* = 14)	0	7 (6.7%)	4 (3.8%)	0	3 (2.9%)
GG3 (*n* = 29)	0	11 (11%)	9 (8.7%)	5 (4.8%)	4 (3.8%)
GG4 (*n* = 24)	0	5 (4.8%)	8 (7.7%)	6 (5.8%)	5 (4.8%)
GG5 (*n* = 23)	0	3 (2.9%)	8 (7.7%)	2 (1.9%)	10 (9.6%)
TB (*n* = 107)	0	37 (35%)	33 (31%)	13 (12%)	24 (22%)
GG1 (*n* = 2)	0	1 (0.9%)	0	0	1 (0.9%)
GG2 (*n* = 28)	0	21 (20%)	7 (6.5%)	0	0
GG3 (*n* = 31)	0	8 (7.5%)	16 (15%)	4 (3.7%)	3 (2.8%)
GG4 (*n* = 27)	0	5 (4.7%)	6 (5.6%)	7 (6.5%)	9 (8.4%)
GG5 (*n* = 19)	0	2 (1.9%)	4 (3.7%)	2 (1.9%)	11 (10%)
SB + TB (*n* = 113)	0	39 (34%)	37 (33%)	13 (12%)	24 (21%)
GG1 (*n* = 0)	0	2 (1.8%)	1 (0.9%)	0	0
GG2 (*n* = 7)	0	14 (12%)	4 (3.5%)	0	0
GG3 (*n* = 13)	0	12 (11%)	13 (12%)	2 (1.8%)	1 (0/9%)
GG4 (*n* = 23)	0	9 (8.0%)	10 (8.8%)	8 (7.1%)	5 (4.4%)
GG5 (*n* = 26)	0	2 (1.8%)	9 (8.0%)	3 (1.7%)	18 (16%)

*Abbreviations*: *SB* Systematic biopsy, *TB* Target biopsy, *GG* Gleason grade

GG concordance between SB and RP specimens was observed in 32 cases (31%), with 37 (36%) and 35 cases (34%) being underestimated and overestimated, respectively (weighted kappa coefficient: 0.24). Grade concordance between TB and RP specimens was found in 55 cases (52%), with 25 (23%) and 27 cases (25%) being underestimated and overestimated, respectively (weighted kappa coefficient: 0.47). Grade concordance between SB + TB and RP specimens was found in 53 cases (47%), with 15 (13%) and 45 cases (40%) being underestimated and overestimated, respectively (weighted kappa coefficient: 0.44).

## Discussion

In the diagnosis of prostate cancer, it is crucial to identify clinically significant prostate cancers that would benefit from treatment [12]. Several reports have suggested that MRI-TRUS fusion biopsy is superior in detecting clinically significant prostate cancer (csPCa) because it can accurately assess lesions noted on MRI [13–16]. PI-RADS evaluation has become standard to interpret MRI and mpMRI is used, which combines anatomic T2W imaging with functional and physiologic assessment, including diffusion-weighted imaging (DWI) and its derivative apparent-diffusion coefficient (ADC) maps, dynamic contrast-enhanced (DCE) MRI [9, 17]. High detection rates of csPCa have been reported for PI-RADS category 4 and 5 lesions, making them suitable candidates for targeted biopsy. However, the detection rate of cancer in category 3 lesions varies from 5 to 26%, depending on the report, and management of these lesions has not been established [18–21]. At our institution, PI-RADS category ≥ 3 is the target for targeted biopsy.

Due to the growing demand for prostate diagnostics, it is imperative to address the long waiting time for mpMRI and the burden on radiologists [22, 23]. Additionally, cost reduction is necessary where possible. To tackle these challenges, one potential solution is MRI without gadolinium-based contrast agents (bpMRI). Nonenhanced MRI can improve patient throughput by reducing examination time and the amount of MRI preparation required prior to the examination, including precautions regarding contrast media. Additionally, MRI protocols that do not require the injection of contrast agents are preferred by patients, which can reduce patient discomfort and side effects (e.g., hematoma, contrast extravasation, allergic reactions, nephrogenic systemic fibrosis in patients with impaired renal function, intracranial gadolinium deposition), while also reducing time in the scanner [24]. Most MRI studies of suspected cancer can also be identified using only T2-weighted MRI and DWI criteria, as can a significant proportion of large tumors and PI-RADS 4 lesions, especially those assigned to the PI-RADS 5 category. DCE-MRI can be useful in detecting small cancers that are less prominent or occult on T2-weighted images and DWI, or when DW images are affected by prostheses [9, 25]. Local contrast enhancement increases the confidence of the reader and helps inexperienced readers find MRI-positive scans [26–28]. A UK-based study reported that the addition of DCE-MRI to T2W and DWI led to a significant increase in overall cost, approximately 70%, due to the inclusion of contrast media, syringes, scanner time, and reading times. However, the clinical benefit of this additional cost is not clear, and further research is needed to determine whether the added benefit justifies the extra expense [29]. Schoots et al. reported that the PI-RADS committee needs better quality data to make evidence-based recommendations for contrast-free MRI as an initial diagnostic approach to prostate cancer screening [30]. In this study, we examined the accuracy of bpMRI-TRUS fusion biopsy by evaluating postoperative pathology specimens. The reported sensitivity of mpMRI for detecting IT ranged from 76–93% [31, 32]. Baco et al. reported the accuracy of histologically confirmed IT detection with mpMRI-TRUS fusion biopsy as 95% (n = 135), while Francesco et al. reported it as 82% (*n* = 152) [5, 33]. In this study, which used bpMRI-TRUS fusion biopsy, the accuracy of IT assessment was 86% (97/113), which we considered to be comparable to previous mpMRI reports. Furthermore, Baco et al. reported that mpMRI-assessed ITs underestimate tumor volume by 5.9 [5].In our study, the maximum IT diameter assessed by bpMRI was also underestimated, with a mean of 11.8 mm on MRI compared to 17.7 mm on RP specimens, although no figure was provided. In terms of T stage, bpMRI and RP specimens were consistent in approximately half of the cases. cT2a cases were upgraded to cT2c in 25 of 32 cases, while cT3a cases were downgraded to cT2 in 28 of 41 cases. This finding suggests that RP specimens may reveal microlesions that are undetectable on MRI and that evaluating micro extracapsular invasion can be challenging. Ploussard et al. reported a concordance rate of GS between TB alone and RP samples in mpMRI/TRUS fusion biopsy of 45% [34], which is similar to the 52% concordance rate for TB alone found in this study. However, the GS concordance rate between SB + TB and RP samples increased to 52% in Ploussard et al.'s study, whereas in our study, it decreased to 47%. In our study, TB alone had the highest accuracy of GS concordance with the RP specimen, but the addition of SB reduced the rate of preoperative underestimation from 23 to 13%. Although the reason for this is unclear, the results suggest that bpMRI does not confer inferiority in GS evaluation, at least in the case of TB alone. The question of whether to omit SB and the optimal number of SB cores remain controversial [35], and the role of the target plus peri-target approach has been recently reported [36].

A limitation of this study is that the cohort included only patients with PI-RADS category ≥ 3 lesions detected by bpMRI who underwent radical prostatectomy. Therefore, this study does not account for cases where the diagnosis of prostate cancer was missed because MRI omitted the gadolinium-enhanced sequences. In addition, the quality of radiological interpretation and the biopsy technique were not verified, and thus, cannot be directly compared with previous reports. Nevertheless, our study provides insights into the usefulness and limitations of bpMRI in the era of increasing use of MRI/TRUS fusion biopsy.

## Conclusion

The diagnostic accuracy of bpMRI/TRUS fusion biopsy is comparable to that of previous reports using mpMRI. Furthermore, the results suggest that bpMRI/TRUS fusion biopsy is useful in terms of saving time and cost. Further research is necessary to verify the cases in which there is no disadvantage in using bpMRI.

## Data Availability

The datasets analyzed during the current study are available from the corresponding author on reasonable request.
